# Hypoprolactinemia as a Clue to Diagnosis of Mild Central Hypothyroidism due to *IGSF1* Deficiency

**DOI:** 10.4274/jcrpe.galenos.2019.2019.0085

**Published:** 2020-06-03

**Authors:** Anastasios Papadimitriou, Anna Papadopoulou, Kleanthis Kleanthous, Dimitrios T. Papadimitriou, Vassiliki Papaevangelou

**Affiliations:** 1 National and Kapodistrian University of Athens, “Attikon” University Hospital, Third Department of Pediatrics, Athens, Greece

**Keywords:** Central hypothyroidism, hypoprolactinemia, IGSF1

## Abstract

Loss-of-function mutations of *IGSF1* are an X-linked cause of central hypothyroidism (CeH) and hypoprolactinemia. A boy who is now 15.2 years old presented at the age of 7.69 years for evaluation of obesity. Previous thyroid function evaluation suggested CeH [FT4 0.6 ng/mL, thyroid-stimulating hormone (TSH) 2.2 mIU/L] but his physician took no action. At presentation he was clinically and biochemically euthyroid, prepubertal and obese. Serum prolactin (PRL) was undetectable. Biochemistry was normal except for mild hypercholesterolemia, total cholesterol 198 mg/dL. Subsequently FT4 and TSH levels fluctuated between 0.72-0.95 ng/dL (normal 0.8-2.0) and 1.94-5.77 mIU/L (normal 0.3-5.0), respectively. Sequencing of *IGSF1* gene revealed a novel genetic change c.3805C>T in exon 19; substitution of amino acid Arginine at position 1269 with a premature «stop» codon resulting in an altered protein product. The patient additionally presented delayed adrenarche, low height velocity that resolved spontaneously and normal pubertal onset associated with increased FSH levels. At 14 years-of-age, while the patient was at Tanner stage 4, PRL levels became detectable, rising gradually to 2.3 ng/mL at last examination. Thyroxine replacement therapy resulted in decrease in total cholesterol 103 mg/dL. A high index of suspicion for the disorder is needed since several measurements of thyroid function may be required for CeH to be disclosed. The patient’s normal FT4 levels and normal intelligence would have resulted in a missed diagnosis if the serum PRL levels had not been measured. This case highlights the importance of measuring PRL in a boy with low normal FT4 and normal TSH levels.

**What is already known on this topic?**

IGSF1 deficiency has been recently found to be an X-linked cause of central hypothyroidism (CeH). Additionally patients may present hypoprolactinemia, obesity, transient partial growth hormone deficiency, delayed adrenarche, normal timing of testicular enlargement but delayed testosterone rise in puberty resulting in delayed adolescent growth spurt, and adult macro-orchidism.

**What this study adds?**

We present an asymptomatic boy with mild CeH due to a novel mutation of *IGSF1* gene. He mostly had low normal FT4 levels while PRL was undetectable. If he had not had his PRL levels measured most probably diagnosis would be missed.

## Introduction

Loss-of-function mutations of the immunoglobulin superfamily, member 1 *(IGSF1)* gene have been recently described as an X-linked cause of congenital central hypothyroidism (CeH) (1), with an estimated prevalence of 1/100000 (2). CeH is the hallmark of the disorder, however, patients additionally may present with hypoprolactinemia, transient partial growth hormone (GH) deficiency (GHD), normal timing of testicular enlargement but delayed testosterone rise in puberty resulting in delayed adolescent growth spurt, and adult macro-orchidism (3). The *IGSF1* gene resides on the X-chromosome and thus its mutations affect mainly males, although female heterozygous carriers may present with CeH (3). The prevalence of low FT4 in female carriers is reported to be 18% (4). The *IGSF1* gene encodes an immunoglobulin superfamily glucoprotein of the plasma membrane and the *IGSF1* protein was observed in somatotropes, thyrotropes, and lactotropes of anterior pituitary, whereas it was absent in gonadotropes or corticotropes. Moreover, the *IGSF1* protein is predominantly expressed in testis, muscle, heart and pancreas.

We present a boy with mild CeH due to a novel mutation of the *IGSF1* gene. Additionally, the patient presented with undetectable prolactin (PRL) levels, that proved to be the clue to diagnosis.

## Case Report

A boy of Greek descent, who is currently 15.2 years old, presented to our pediatric endocrinology clinic at the age of 7.69 years for obesity evaluation. He is the first child of unrelated parents, born after normal delivery with normal body weight and length. Developmental milestones were achieved at a normal age. During the preschool years he had normal height velocity but increase in body weight. Thyroid function tests (TFT) ordered by his pediatrician, at 3 and 4 years-of-age, were compatible with CeH (FT4 0.5 ng/mL, thyroid-stimulating hormone (TSH) 2.2 mIU/L and FT4 0.65 ng/mL, TSH 1.8 mIU/L, respectively), however, no action was taken. His parents and siblings (a girl and twin boys currently 13 and 9.5 years old, respectively) are healthy. Mother did not breast-feed any of her four children because of inadequate milk production.

At presentation, the patient’s height standard deviation score (HSDS) was 122.5 cm (HSDS -0.55). He was prepubertal and euthyroid, with no typical symptoms of hypothyroidism such as fatigue, constipation, or bradycardia. His weight  was 35.1 kg (WSDS 1.67), body mass index (BMI) 23.4 kg/m2 (BMI-SDS 2.89). The thyroid gland was non-palpable. School performance was reported as very good. Target height (TH) SDS was +1.1.

TFT showed FT4 1.0 ng/dL (0.8-2.0), TSH 1.98 mIU/L (0.3-5.0), PRL <0.7 ng/mL (3-18), insulin like growth factor 1 (IGF1) 126 ng/mL (110-565) and bone age was 6.7 years. Biochemistry was normal except for a mild increase in total cholesterol 198 mg/dL (<170), high-density lipoprotein (HDL)-cholesterol 68 mg/dL (>40), low-density lipoprotein (LDL)-cholesterol 123 mg/dL (<129) and triglycerides 36 mg/dL (<150). During the next two years there was fluctuation of FT4 levels between 0.72-0.95 ng/dL, of TSH levels between 1.94-5.77 mIU/L, whereas PRL was always undetectable. Thyrotropin releasing hormone (TRH) test showed a normal TSH response, 0’:3.44 mIU/L, 30’:14.73 mIU/L, 60’:11.71 mIU/L, and an abnormal PRL response 0’: <0,4 ng/mL, 30’: 1.7 ng/mL, 60’: 0.9 ng/mL. Basal PRL levels became detectable at 1.7 ng/mL at the age of 14 years, at Tanner stage 4, increasing slightly to 2 ng/mL and 2.3 ng/mL at the age of 14.7 years and 15.2 years, respectively. Thyroid ultrasonography revealed a hypoplastic thyroid gland, total thyroid volume 2.1 mL and 2.2 mL at 9 and 15.2 years-of-age, respectively.

**Figure 1:**
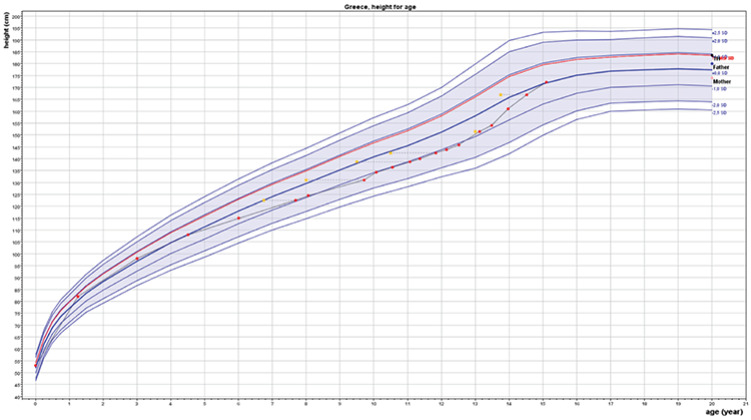
Progression of height. Height velocity normalized spontaneously after the age of 10 years. Squares denote bone ages. Arrow depicts initiation of L-thyroxine treatment

At 9.8 years, due to low height velocity (Figure 1), a GH stimulation with glucagon was performed that showed a subnormal peak level of serum GH 4.7 ng/mL and normal peak serum cortisol 25.7 µg/dL (normal >18 µg/dL). Serum IGF1 was 116 ng/mL (normal 110-565). Brain MRI showed a normal pituitary. Soon afterwards the boy presented with spontaneous normalization of height velocity and we therefore suspended further testing of the GH axis. At the age of 11 years thyroxine replacement was started, his FT4 being a little below normal 0.7 ng/dL, which resulted in undetectable TSH. Normalization of FT4 had no substantial difference in the boy’s general well-being nor in his growth parameters (height, BMI), however it was associated with a substantial decrease in lipid levels, total cholesterol 103 mg/dL, HDL-cholesterol 51 mg/dL, LDL-cholesterol 45 mg/dL, triglycerides 37 mg/dL.

The boy entered puberty at 12 years of age. FSH levels were increased at 9.3 to 11.4 mIU/mL, during prepuberty and early puberty, whereas LH levels were normal. At last examination, at the age of 15.2 years serum FSH was still increased (15.3 mIU/mL) with normal testicular volume (TV) of 18 mL. Testosterone levels were <10 ng/mL until TV 12 mL. At last examination testosterone levels were 451 ng/dL, being low normal for a TV of 18 mL. Moreover, the boy presented with delayed biochemical adrenarche [serum dehydroepiandrosterone sulfate (DHEAS) being 12 ng/mL at the age of 7.8 years, 16.1 ng/mL at the age of 9.9 years, 36 ng/mL at the age of 11.8 years and 75 ng/mL at 12.5 years of age] and delayed pubarche at 13 years of age. The patient also exhibited a delayed onset of pubertal growth spurt, at about 13 years of age when TV was 13.5 mL.

Height velocity is normal, as are serum IGF1 levels at 316 ng/mL (152-540), and predicted adult height is within TH.

TFT of his mother showed normal levels of T4 6.24 µg/dL, TSH 2.97 mIU/L, as well as PRL 8.5 ng/mL. His twin brothers also had normal TFT and PRL, brother 1: FT4 1.34 ng/dL, TSH 2.80 mIU/L, PRL 3.6 ng/mL and brother 2: FT4 1.44 ng/dL, TSH 2.87 mIU/L, PRL 4.5 ng/mL.

### Molecular Analysis

**Figure 2:**
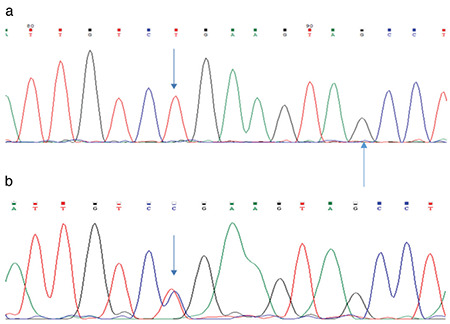
Sequencing of the *IGSF1* gene showing the c.3805C>T (p.Arg1269Ter) genetic change in exon 19: (a) patient, (b) mother

Analysis of *IGSF1* gene revealed a genetic change, c.3805C>T in exon 19 (Figure 2), that resulted in substitution of amino acid Arginine at position 1269 with a «stop» codon and the production of an altered protein product. This genetic change has not been reported previously in patients with CeH. We also performed analysis of the gene in the boy’s mother and sister. His mother was found to carry the same mutation as the proband, but no mutation was found in his sister. *IGSF1* gene analysis was not performed in his brothers because of normal thyroid function in both of them.

## Discussion

We identified a novel *IGSF1* nonsense mutation in a Greek patient with congenital CeH. The molecular defect observed in our patient (p.Arg1269X) prematurely truncates the *IGSF1* protein at the end of the 12^th^ Ig loop in the extracellular portion of the C-terminal domain. The *IGSF1* protein includes 12 Ig-like domains in two sets of five and seven motifs separated by a linker region, followed by a transmembrane domain and a short cytoplasmic tail (5). The N-terminal segment undergoes translational proteolysis while the C-terminal is expressed extracellularly at the plasma membrane. The precise molecular role of *IGSF1* remains unclear.

To date, more than 30 distinct mutations have been described including missense, nonsense, frameshift and whole gene deletions (6,7) that lead to loss of protein function. All but one of the reported mutations are located in the C-terminal domain of the protein and impair *IGSF1* trafficking from the endoplasmic reticulum to the plasma membrane. There is no clear genotype-phenotype correlation, while variation in the extent of hypothyroidism or the other clinical features, even within families, has been reported (8,9).

*IGSF1* is expressed in thyrotrope cells of the anterior pituitary. *IGSF1*-deficient male mice have reduced serum TSH and decreased pituitary Trhr mRNA levels (1), while others have shown that the principal impairment is attenuated TRH actions in pituitary thyrotropes (10). Garcia et al (11), in a patient with severe congenital CeH due to complete deletion of the *IGSF1* gene, described markedly decreased TSH bioactivity, poor response to TRH stimulation and decreased TRHR expression. Our patient showed a normal TSH response to TRH stimulation, suggesting impaired endogenous TRH action. Moreover, he had a hypoplastic thyroid gland, a finding observed in 74% of *IGSF1* deficient patients (4).

*IGSF1* protein is detected in pituitary lactotropes, however PRL deficiency is present in about 67% of *IGSF1*-deficient patients (3). No explanation for normal PRL levels has been given. Our patient had undetectable serum PRL, and very poor PRL response to TRH stimulation suggesting pituitary dysfunction. However, basal PRL levels became detectable at the age of 14 years showing a gradual increase. It remains to be seen whether PRL will normalize as the child grows older.

Increased birth weight or length is observed in a substantial number of patients (12). 67% of *IGSF1*-deficient male children were classified as overweight and 21% as obese (4), being in accord with the phenotype of our patient. It is unclear how these metabolic alterations are related to *IGSF1* deficiency.

Children with *IGSF1* deficiency present with disharmonious pubertal development, that is pubertal onset at a normal age but delayed testosterone increase occurring at an advanced TV. In adult life testosterone levels are usually low or low normal. Late adolescent and adult patients commonly present with macro-orchidism, however, TV may be normal (13) or increased from the prepubertal years. Our patient entered puberty at a normal age. At onset of puberty the patient’s basal FSH levels were increased, LH concentrations were normal for pubertal onset and showed a normal progression according to pubertal status, whereas testosterone levels in early puberty were low for TV but normalized as puberty progressed. It is not clear what causes the disharmonious pubertal development in these patients.

Patients with *IGSF1* deficiency have been reported to present with delayed adrenarche (14). Our patient presented with the marker of biochemical adrenarche, that is serum DHEAS ≥40 µg/dL, after the age of 12 years and pubarche at the age of 13 years. The median age of pubic hair development for Greek boys is 11.2 years (15).

Transient partial GH deficiency has been reported in a subset of patients with *IGSF1* deficiency. It is not clear why our patient exhibited growth deceleration, although subnormal GH secretion, low normal IGF1 levels and the delayed bone age might suggest transient GH deficiency that resolved before adolescence. The period between 6 and 11 years of age in boys constitutes the juvenile phase of growth characterized by growth deceleration relative to the preceding childhood phase and by increase of adrenal androgens (adrenarche) (16). Based on the very low DHEAS levels of our patient during this period we can speculate that low adrenal androgens may exaggerate the normal growth-decelerating pattern of the juvenile period. Normalization of height velocity, which occurred prior to thyroid hormone substitution, might be attributed to the gradual increase of adrenal androgens.

## Conclusion

In conclusion, we present a male patient with CeH and PRL deficiency due to a novel mutation of the *IGSF1* gene. Additionally, he presented with obesity, disharmonious puberty, and delayed adrenarche which are all features of the *IGSF1* syndrome. The patient had mostly low normal FT4 levels, thus PRL deficiency was the clue to diagnosis. Most reported cases of CeH due to *IGSF1* deficiency are symptomatic necessitating L-thyroxine replacement. We believe, however, that a significant number of patients are undetected because symptoms may be absent or subtle. Diagnosis is important for genetic consultation, since no clear genotype-phenotype correlation is observed, even within the same family. Furthermore, with TSH-based neonatal congenital hypothyroidism screening programs neonatal diagnosis will be missed and definitive diagnosis is likely to be delayed. Children of female carriers and female children of male patients should be screened in neonatal life for FT4 and TSH levels. This case highlights the importance of determining PRL levels in a boy with low normal FT4 and normal TSH levels.
